# Real‐world data on lenalidomide dosing and outcomes in patients newly diagnosed with multiple myeloma: Results from the Canadian Myeloma Research Group Database

**DOI:** 10.1002/cam4.5245

**Published:** 2022-09-26

**Authors:** Hira Mian, Richard LeBlanc, Martha Louzada, Esther Masih‐Khan, Arleigh McCurdy, Christopher P. Venner, Julie Stakiw, Moustafa Kardjadj, Victor H. Jimenez‐Zepeda, Michael Sebag, Darrell White, Muhammad Aslam, Kevin Song, Anthony Reiman, Rami Kotb, Engin Gul, Donna Reece

**Affiliations:** ^1^ McMaster University Hamilton Ontario Canada; ^2^ Maisonneuve‐Rosemont Hospital Research Centre University of Montreal Montreal Quebec Canada; ^3^ London Regional Cancer Center London Ontario Canada; ^4^ Canadian Myeloma Research Group Vaughan Ontario Canada; ^5^ Princess Margaret Cancer Centre Toronto Ontario Canada; ^6^ The Ottawa Hospital Ottawa Ontario Canada; ^7^ Cross Cancer Institute University of Alberta Edmonton Alberta Canada; ^8^ Saskatoon Cancer Centre University of Saskatchewan Saskatoon Saskatchewan Canada; ^9^ Arnie Charbonneau Cancer Institute University of Calgary Calgary Alberta Canada; ^10^ McGill University Montreal Quebec Canada; ^11^ Queen Elizabeth II Health Sciences Centre Dalhousie University Halifax Nova Scotia Canada; ^12^ Allan Blair Cancer Centre Regina Saskatchewan Canada; ^13^ BC Cancer Agency Vancouver General Hospital Vancouver British Columbia Canada; ^14^ Saint John Regional Hospital Saint John New Brunswick Canada; ^15^ Cancer Care Manitoba Winnipeg Manitoba Canada

**Keywords:** dosing, lenalidomide, multiple myeloma, transplant‐ineligible

## Abstract

Using the Canadian Myeloma Research Group Database, a retrospective study of 167 newly diagnosed, transplant‐ineligible patients with multiple myeloma (MM) that received lenalidomide‐dexamethasone as front‐line treatment was conducted to understand the impact of lenalidomide dosing. Starting dose modifications were common, 42% of patients started on lenalidomide <25 mg with normal renal function. During treatment course, 35% of patients required further dose reduction. Dose reductions in the first year did not have an impact on progression free survival or overall survival. Further studies need to be conducted to understand the impact of dosing strategies of anti‐MM agents in the real world.

## 1 INTRODUCTION

Multiple myeloma (MM) is an incurable plasma cell neoplasm that predominantly affects older adults with a median age of 69 years.^1^ The immunomodulatory drug, lenalidomide, represents a cornerstone of regimens for newly diagnosed transplant‐ineligible patients (TI NDMM) based upon pivotal studies including the FIRST trial, SWOG S0777, and more recently the MAIA trial.^2^, ^3^, ^4^ Although lenalidomide‐containing regimens are credited with improved outcomes, dose reductions and/or interruptions are known to occur even among patients that meet eligibility for clinical trial participation.^5^, ^6^ While novel dosing strategies are being tested in clinical trials,^7^ there is inadequate data describing dose modification decisions made by clinicians in the real‐world. Furthermore, it is unknown how these real‐world dosing strategies and modifications impact patient outcomes. As lenalidomide‐dexamethasone combination continues to form the backbone of many novel regimens, the objective of our study was to understand the dosing, efficacy, and tolerability of lenalidomide among TI NDMM patients in the real‐world setting.

## 2 PATIENTS AND METHODS

We conducted a retrospective review using the national, multi‐center Canadian Myeloma Research Group Database (CMRG‐DB). Local research ethics boards at every contributing site approved data entered into the CMRG‐DB and all patients provided a written or implied informed consent. The approval for this review was from the Hamilton Integrated Research Ethics Board (HiREB) as per the approved governance of the CMRG Database. Consecutive non‐trial TI NDMM patients treated between Jan 2008‐Dec 2018 were included. All patients received lenalidomide 5–25 mg mostly on days 1–21 and dexamethasone 8 – 40 mg on a once weekly schedule (subsequent dexamethasone dosing changes were not captured for this study). Patients were divided based on the starting dose of lenalidomide (25 mg or < 25 mg). Additionally, as product monograph recommendations suggest full dose lenalidomide (25 mg) for estimated glomerular filtration rate (eGFR) ≥ 60 ml/min/1.73 m^2,^ ^8^ we further stratified patients within each dose category (25 mg or < 25 mg) by renal function (eGFR <60 [impaired renal function] or ≥ 60 ml/min/1.73 m^2^ [normal renal function]). Retrospective chart reviews were conducted to assess for causes of dose modification (all grade toxicity). Overall survival (OS) and progression free survival (PFS) were determined and further stratified by starting dose (25 mg versus <25 mg) and renal function (eGFR <60 versus ≥60) using Kaplan–Meier method.

To assess the impact of factors associated with dose reductions, a multivariate logistic regression analysis was conducted. Model fitness was verified with the Hosmer and Lemeshow test, and collinearity between independent variables was verified by correlation analysis. To evaluate the impact of dose reduction in the first year and curtail the effect of early mortality, a landmark analysis was done for PFS and OS at 12 months following therapy initiation. Log‐rank test was used to compare those who received a dose reduction versus no reduction. Statistical analyses were performed using R (4.1.0) and RStudio (1.4.1717) for Windows. All tests were 2‐sided and *p* < 0.05 was considered statistically significant.

## 3 RESULTS

A total of 167 TI NDMM patients receiving Rd in first‐line treatment, not on a clinical trial were identified in our database. Baseline characteristics of these patients are shown in Table 1. The median age was 77 years and 56% were males. High risk cytogenetics were present in 22.6% of the evaluable patients. Anemia was present in 43.4%; however, neutropenia and thrombocytopenia were only present in 0.7% and 6.4% of the cohort. Impaired renal function (eGFR <60) was present in 41.4% of patients. Overall, the median time from diagnosis to Rd initiation was 0.7 months (range 0.1–92.5).

**TABLE 1:** Baseline characteristics and dose modifications in transplant ineligible NDMM patients treated with lenalidomide‐dexamethasone

Characteristics	All patients *N* = 167	Starting dose 25 mg *N* = 63^a^	Starting dose <25 mg *N* = 102^a^
Age, years median (range)	77 (56–94)	74 (56–93)	80 (59–94)
Male gender, *n* (%)	93 (56.0)	43 (68)	49 (48)
Myeloma isotype, *n* (%)^a^			
IgG	94 (57.7)	39 (62.9)	54 (54.6)
IgA	28 (17.2)	10 (16.1)	18 (18.2)
Light chain only	38 (23.2)	12 (19.4)	25 (25.3)
Other	3(1.8)	1 (1.6)	2 (2.0)
Unknown or missing	4	1	3
ISS Staging, *n* (%)^a^			
Stage I	25 (20.2)	16 (32.7)	9 (12.2)
Stage II	49 (39.5)	21 (42.9)	28 (37.8)
Stage III	50 (40.3)	12 (24.5)	37 (50)
Unknown, *n*	43	14	28
Cytogenetic risk, *n* (%)^a^			
High Risk^b^	15 (22.6)	7 (30.4)	8 (20.0)
Standard Risk	49 (77.4)	16 (69.6)	32 (80.0)
Unknown, *n*	103	40	62
Cytopenias at diagnosis, *n* (%)			
ANC <1.0 × 10^9^/L	1 (0.7)	0	1 (1.0)
Plts <100 × 10^9^/L	9 (6.4)	2 (3.2)	8 (8.5)
Hb < 100 g/L	69 (43.4)	24 (38.7)	44 (46.8)
eGFR at diagnosis			
(ml/min/per 1.73 m^2^), *n* (%)			
≥ 60	81 (58.3)	42 (87.5)	38 (42.2)
< 60	58 (41.7)	6 (12.5)	52 (57.8)
Unknown, *n*	28	15	12
Dose increased (*n*, %)	5 (3.0)	0	5 (5.0)
Dose decreased (*n*, %)	58 (35.2)	26 (41.3)	32 (31.7)
No change in dose (*n*, %)	102 (61.8)	37 (59.7)	65 (63.7)
First dose reduction timing, months(median, range)	6.7 (0.8–56.9)	9.4 (0.8–56.9)	4.8 (0.9–35.9)
Tx duration, months (median, range)	17.9 (0.1–113.2)	22.8 (1.3–60.6)	16.0 (0.1–113.2)

Abbreviations: ANC, absolute neutrophil count; eGFR, estimated glomerular filtration rate; Hb, hemoglobin; ISS, international staging system; NDMM, newly diagnosed multiple myeloma; Plts, platelets.

^a^ Starting dose unknown for 2 patients.

^b^ High risk cytogenetics defined as del 17p, t(4;14) and/or t(14;16).

Starting dose, dose modification details, and treatment duration data were available for 165 patients out of 167 and are shown in Table 1. Among the 165 patients, 63 (38.2%) were started on a dose of 25 mg and 102 (62.0%) on <25 mg. The most common dosages among those reduced to <25 mg were 15 mg in 42 patients (25.8%) and 10 mg in 49 patients (30.1%) (Table S1). Among patients started on 25 mg, 87.5% had normal renal function whereas among those started on <25 mg, 42.2.% (*n* = 38) had normal renal function. After adjusting for sex, cytogenetics and staging, patients with impaired renal function (eGFR <60) and those ≥75 years had a 5.78 (95% CI 2.14–17.68, *p* > 0.001) and 5.14 (95% CI 2.33–11.9, *p* > 0.001) increased odds of a reduced starting dose compared to patients with normal renal function (eGFR ≥60) and patients <75 years.

During therapy, dose reductions were present in 35.2% of the overall cohort. Patients started on 25 mg and < 25 mg, 41.3% (*n* = 26) and 31.7% (*n* = 32) required further dose reduction, respectively. Seven patients had their lenalidomide dose reductions by changing the dosing schedule from every day to every other day. The median time to first dose reduction in the overall cohort was 6.7 months (range 0.8–56.9 months). Reasons for dose reduction were available in 38.0% patients. Most frequent toxicities reported in more than 20% of the patients were fatigue (31.6%), neutropenia (29.8%), diarrhea (22.8%), rash (22.8%) and thrombocytopenia (21.1%). In the total cohort, eighteen (10.8%) patients discontinued treatment at least 2 months (range 2.3–25.1) prior to progression for reasons of toxicity (7.2%), palliation (1.2%) and patient decision (2.4%). Conversely, dose escalation was uncommon and only five (3%) of the patients started on <25 mg had their dose increased during therapy. The median age for these five patients was 78 years (range 72–80 years). For 4 patients, the initial dose was increased within 3 months with one patient increasing the dose after 2.4 years.

For the total cohort, the median follow‐up was 33.2 months (range 0.5–113.2). The median PFS and OS was 21.0 (95% CI 14.9–27.4) and 55.5 months (95% CI 45.7–84.0), respectively. The overall response rate (≥ PR) was 78.7% and ≥ VGPR was 52.0%. With respect to dose, the median PFS and OS for patients started on 25 mg versus <25 mg was not statistically significant (Figure S1). To further understand the impact of dosing based upon renal function, we stratified patients with eGFR <60 or ≥ 60 based upon starting lenalidomide dose (25 mg or < 25 mg). There were no significant differences for PFS and OS for patients with eGFR <60 and eGFR ≥60 based upon starting dose of 25 mg or < 25 mg in each group (Figure S2 and S3).

**FIGURE 1:**
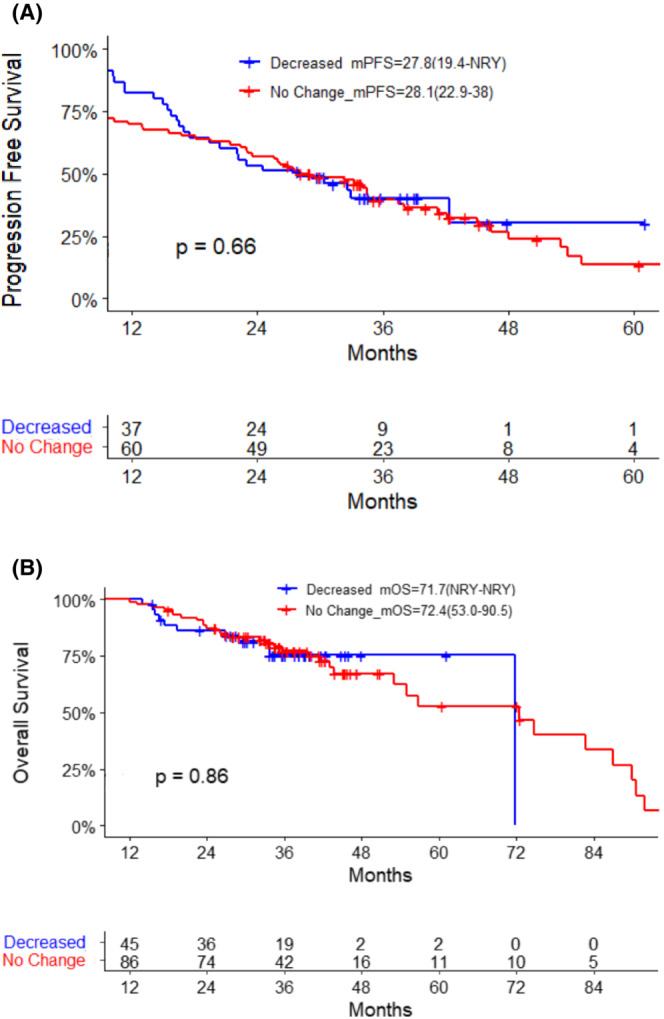
(A) Landmark progressive free survival of patients with dose reduction during year one versus not. (B) Landmark overall survival of patients with dose reduction during year one versus not.

To understand the impact of dose reduction in the first year, a landmark analysis was done for 129 patients alive at 12 months from therapy initiation. The patients included, 94 (73%) had no dose change and 35 (27%) had a change in dose. Both PFS and OS showed no significant differences between those that required dose modification within the first year versus not (Figure 1A,B).

## 4 DISCUSSION

In summary, in this real‐world retrospective study, lenalidomide dose modifications were frequent both at initiation of treatment and during the course of therapy among TI NDMM patients. Dose reduction both at the start of therapy and during the first year following therapy initiation did not influence either PFS or OS.

Regarding dose modifications at therapy initiation, patients that started on a lenalidomide dose less than 25 mg, 42.2% had normal renal function. This normal renal function cohort receiving the starting dose of <25 mg when compared to full dose 25 mg showed a trending difference but no significant difference between both PFS and OS which may be due to the small sample size. Prospective studies with larger number of patients is needed to ascertain if the strategy for initiating therapy with lower lenalidomide doses, for reasons other than renal function is equally as effective. Baseline cytopenias, especially neutropenia, were also uncommon in our cohort suggesting that hematologic disorders were not the cause and additional factors accounted for clinicians selecting a reduced starting dose. While our multivariate analysis identified chronological age > 75 years and impaired renal function as being associated with an increased odds of empiric starting dose reduction, the impact of additional factors including frailty assessment which has emerged as one area for risk stratification was not routinely assessed in our cohort. During the treatment course, 35% of the cohort required dose reductions. This dose reduction in the first year did not significantly impact long term outcomes of PFS and OS suggesting that among older adults who are at high risk of treatment‐related toxicities, treatment discontinuation and overall inferior outcomes,^9^ adapting dosing strategies to balance efficacy and minimize toxicity remain important cornerstones of treatment delivery. Additionally, it was also interesting to note that among those started on lower dose, the more common clinical practice was to maintain the same dose rather than escalating. Future analysis incorporating frailty assessments both at the time of therapy initiation and longitudinally over time should be conducted to further understand the impact of frailty on dosing and overall outcomes in this patient cohort.

Comparing our results to the clinical trials including the FIRST trial show comparable rates of lenalidomide dose reduction which were found to be 37% and 44% among patients on Rd continuous among ≤75 and > 75 years, respectively.^5^ Subsequent efficacy rates of ORR, PFS and OS were also comparable in our cohort to the FIRST trial in which ORR was found to be 81%, median PFS 26.0 months and OS 59.1 months.^2^ With increasing experience using lenalidomide, the outcomes of clinical trials patients on Rd have improved including in the SWOG S0777 and the MAIA trial where the median PFS of 29.0 months and 34.4 months, respectively^3^, ^4^; however, further studies will need to evaluate any incremental benefit over time among non‐trial patients. Additionally, as our cohort was limited to the doublet regimen of Rd which has been the standard of care until recently, the impact of lenaliomide dosing in the more contemporary triplet regimens including lenalidomide‐bortezomib‐dexamethasone (RVd) and daratumumab‐lenalidomide‐dexamethasone (DRd) remains unknown in non‐trial patients.

In the real‐world, there is a paucity of data describing dose reductions and impact on subsequent outcomes among patients on lenalidomide. In the maintenance setting of 10 mg dose, a single center study suggested that over 40% of the individuals were started at a lower dose and over 50% required a dose reduction.^10^ Similarly, in the relapsed setting, real‐world data suggest that contrary to clinical trials most patients are in fact started on a much lower dose of lenalidomide.^11^ Additionally, studies looking at alternative dosing and administration schedules also suggest that the overall efficacy may not be compromised despite these changes.^7^, ^12^, ^13^

In conclusion, our study highlights that although dose modifications based on clinician judgment are common in the real‐world, lenalidomide‐based regimens continue to be effective among TI NDMM patients. Future studies specifically focusing on decision‐making factors as well as prospective studies incorporating frailty assessment in dosing strategies as currently being done in the UK fitness trial^14^ are required to further optimize outcomes among this cohort of patients.

## AUTHOR CONTRIBUTIONS

**Hira Mian:** Conceptualization (lead); data curation (supporting); formal analysis (equal); funding acquisition (equal); investigation (equal); methodology (lead); writing – original draft (lead); writing – review and editing (equal). **Richard LeBlanc:** Conceptualization (supporting); data curation (supporting); formal analysis (equal); investigation (equal); writing – original draft (supporting); writing – review and editing (equal). **Martha Louzada:** Conceptualization (supporting); data curation (supporting); formal analysis (equal); investigation (equal); methodology (equal); writing – review and editing (equal). **Esther Masih‐Khan:** Conceptualization (supporting); data curation (lead); formal analysis (equal); funding acquisition (supporting); investigation (supporting); methodology (supporting); project administration (equal); validation (supporting); writing – review and editing (supporting). **Arleigh McCurdy:** Data curation (supporting); formal analysis (supporting); methodology (lead); writing – review and editing (equal). **Christopher Venner:** Conceptualization (supporting); data curation (supporting); formal analysis (equal); funding acquisition (supporting); investigation (supporting); methodology (equal); writing – review and editing (equal). **Julie Stakiw:** Data curation (supporting); writing – review and editing (equal). **Moustafa Kardjadj:** Formal analysis (lead); writing – review and editing (supporting). **Victor H Jimenez‐Zepeda:** Data curation (supporting); writing – review and editing (equal). **Michael Sebag:** Data curation (supporting); writing – review and editing (equal). **Darrell White:** Data curation (supporting); writing – review and editing (equal). **Muhammad Aslam:** Data curation (supporting); writing – review and editing (equal). **Kevin W Song:** Data curation (supporting); writing – review and editing (equal). **Anthony Reiman:** Data curation (supporting); writing – review and editing (equal). **Rami Kotb:** Data curation (supporting); writing – review and editing (equal). **Engin Gul:** Data curation (supporting); funding acquisition (equal); project administration (equal); writing – review and editing (equal). **Donna Reece:** Conceptualization (supporting); data curation (supporting); formal analysis (equal); funding acquisition (equal); investigation (equal); methodology (supporting); writing – review and editing (equal).

## FUNDING INFORMATION

This work was supported by the Canadian Myeloma Research Group through funds from Celgene Inc.

## CONFLICT OF INTEREST

Mian: Honoraria: Celgene, Janssen, Amgen, Takeda, Sanofi and GSK Awards: HHS Research Early Career Award from Hamilton Health Sciences Foundation. LeBlanc: Membership on an entity's Board of Directors or advisory committees: Celgene Canada; Janssen Inc.; Amgen Canada; Takeda Canada; Sanofi Canada; Research Funding: Celgene. Louzada: Honoraria: Janssen, Celgene, Amgen and Pfizer. McCurdy: Honoraria: Celgene, Janssen, Amgen, Takeda, Sanofi and GSK. Venner: Honoraria: Janssen, Amgen, Takeda; Research Funding: Celgene, Amgen. Jimenez‐Zepeda: Honoraria: Celgene, Janssen, Takeda, Merck and BMS. Sebag: Membership on an entity's Board of Directors or advisory committees: Janssen Inc.; Amgen Canada; Takeda Canada; Celgene Canada. White: Honoraria: Amgen, Antengene, Celgene, Janssen, Karyopharm, Sanofi and Takeda. Song: Research funding: Celgene. Honoraria: Celgene, Janssen, Amgen, Takeda. Kotb: Research funding: Merck, Sanofi. Ownership/Share holder: Karyopharm. Honoraria: Celgene/BMS, Janssen, Takeda, Amgen, Sanofi, Merck. Reece: Research funding: Otsuka, Celgene, Janssen, Takeda, Merck, BMS, Millennium; Consultancy: Celgene, Jansen, Amgen, Karyopharm, Takeda; Honoraria: Celgene Honoraria: Celgene, Janssen, Amgen, Takeda, Sanofi and GSK, Janssen, Amgen, Takeda. All remaining authors have declared no financial or perceived conflicts of interest.

## ETHICS APPROVAL STATEMENT

The approval for this review was from the Hamilton Integrated Research Ethics Board (HiREB), Project Number: 7696‐C.

## PATIENT CONSENT STATEMENT

All patients whose data are reported to the CMRG Database by participating centers have provided informed consent for use of their information for research purposes.

## Supporting information


Figure S1A

Figure S1B

Figure S2A

Figure S2B

Figure S3A

Figure S3B

Table S1

## Data Availability

Canadian Myeloma Research Group database (CMRG‐DB) governance and research ethics boards at sites contributing data do not allow sharing of patient level data. Any reasonable request for aggregate data, supporting the findings of this study can be available from the corresponding author.
