# Supplementary figures and images for: Real‐world data on lenalidomide dosing and outcomes in patients newly diagnosed with multiple myeloma: Results from the Canadian Myeloma Research Group Database
Source: Cancer Med. 2022 Sep 26;12(4):4357–62. doi: 10.1002/cam4.5245 (PMC9972020; doi:10.1002/cam4.5245)

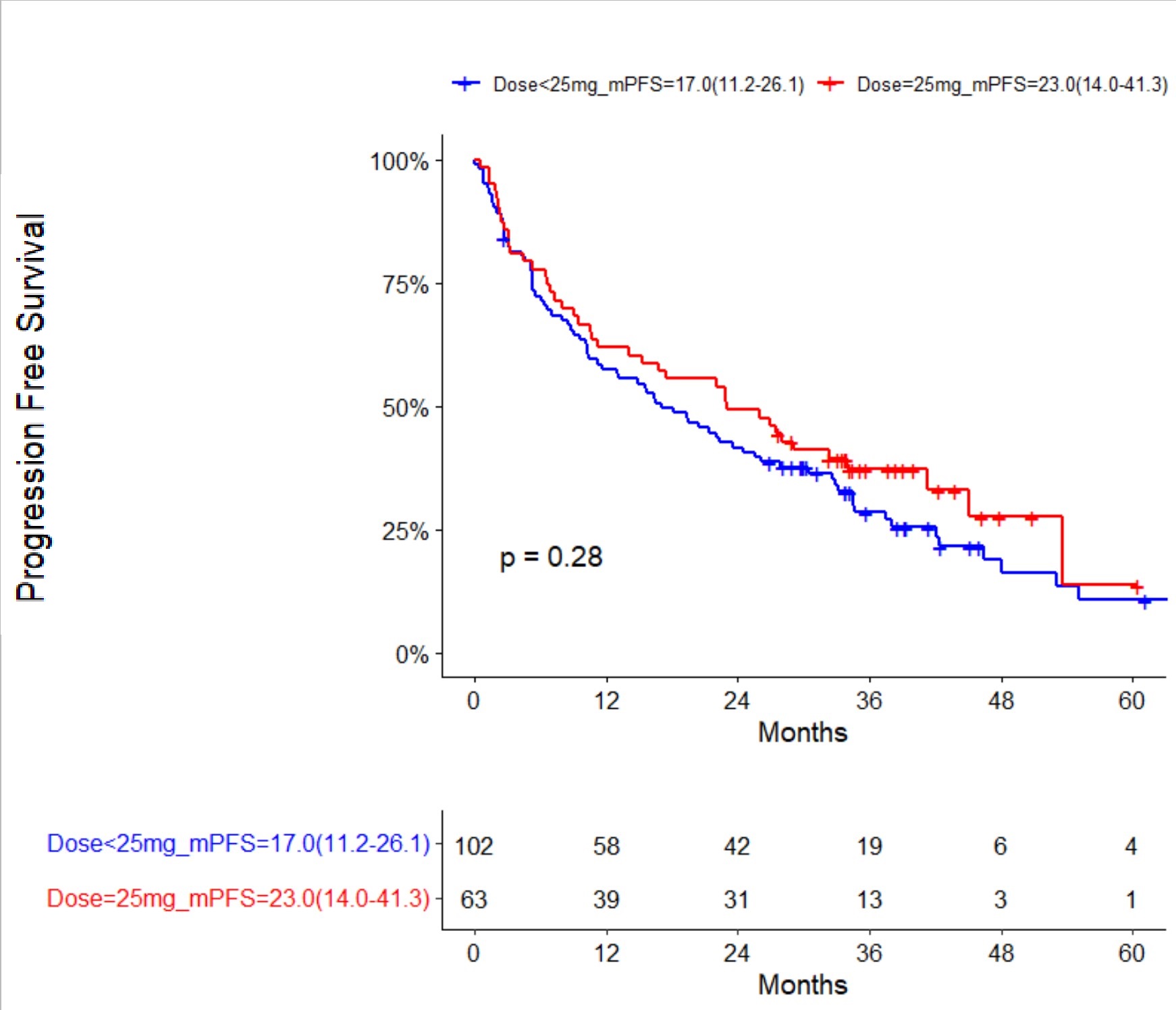

Supplement: Supplementary file 1 — Figure S1A [file CAM4-12-4357-s005.tiff]

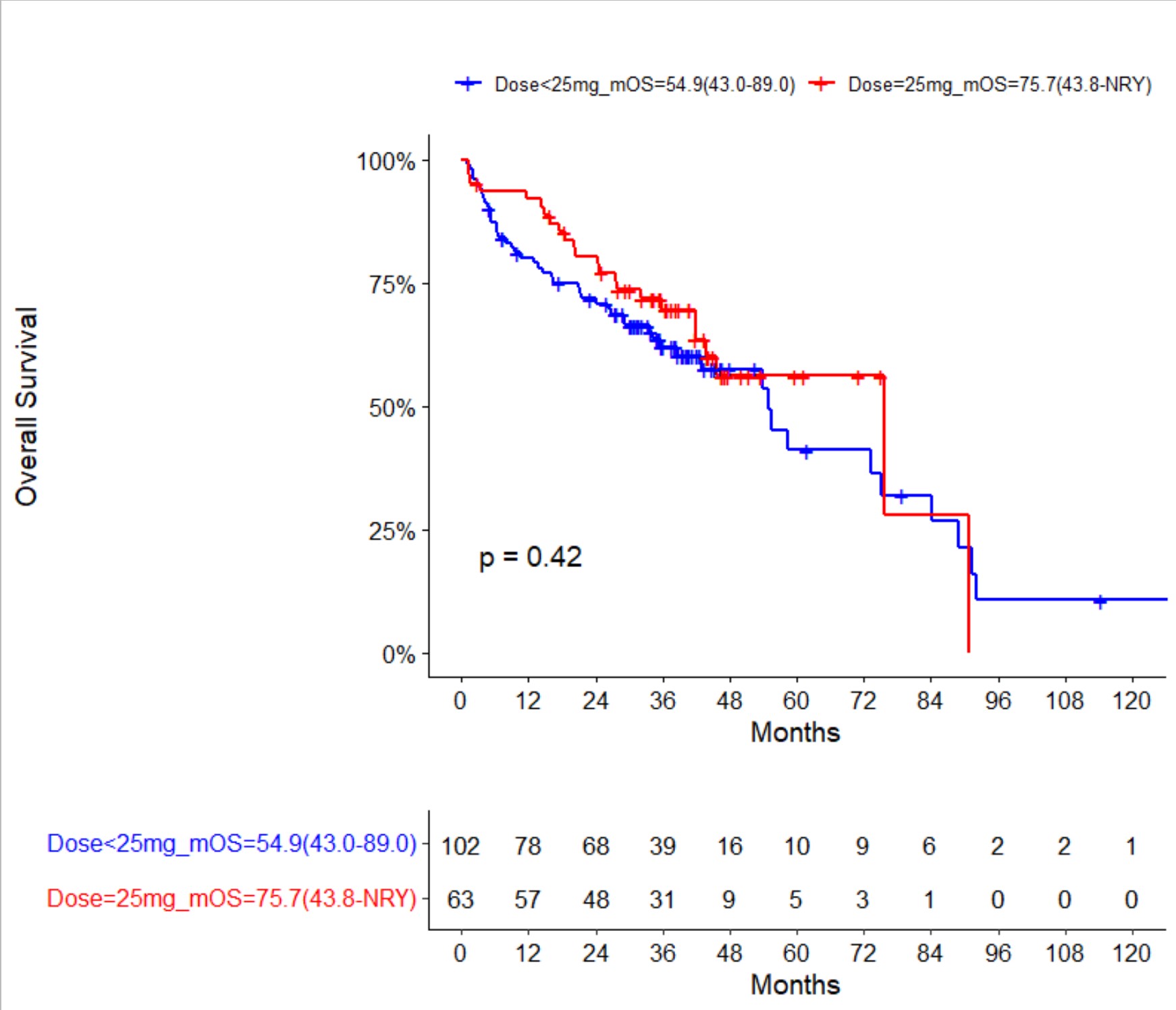

Supplement: Supplementary file 2 — Figure S1B [file CAM4-12-4357-s007.tiff]

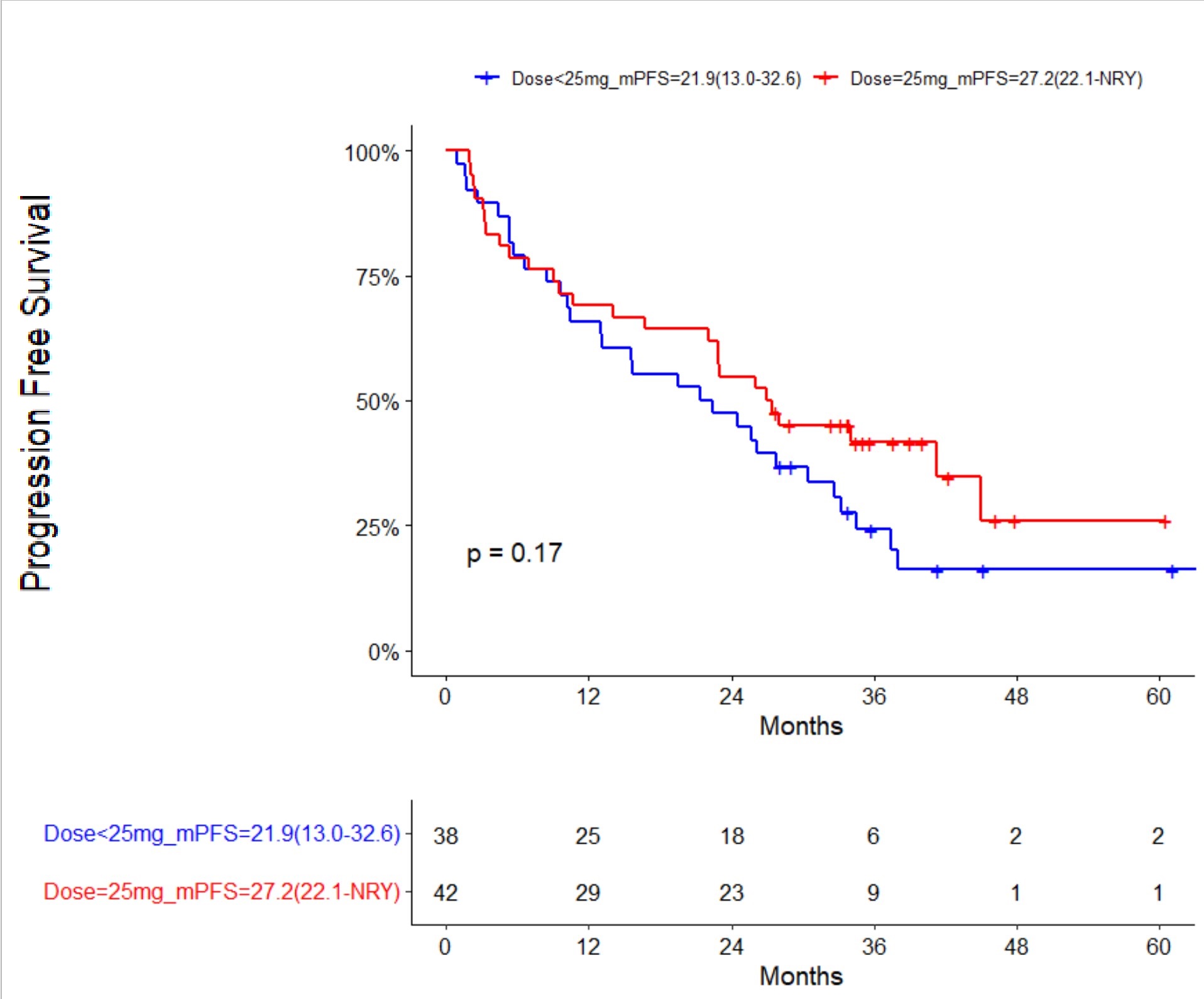

Supplement: Supplementary file 3 — Figure S2A [file CAM4-12-4357-s004.tiff]

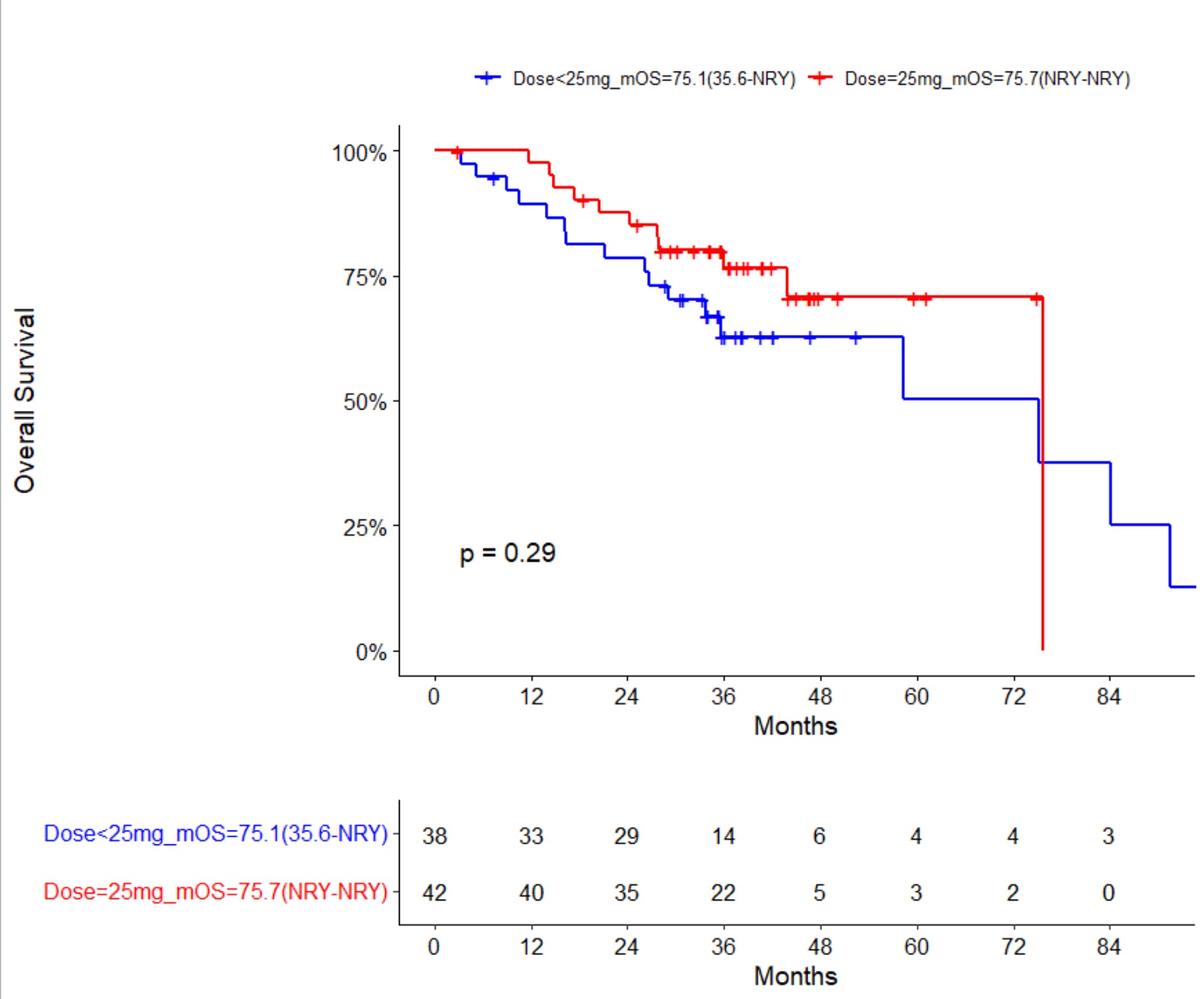

Supplement: Supplementary file 4 — Figure S2B [file CAM4-12-4357-s002.tiff]

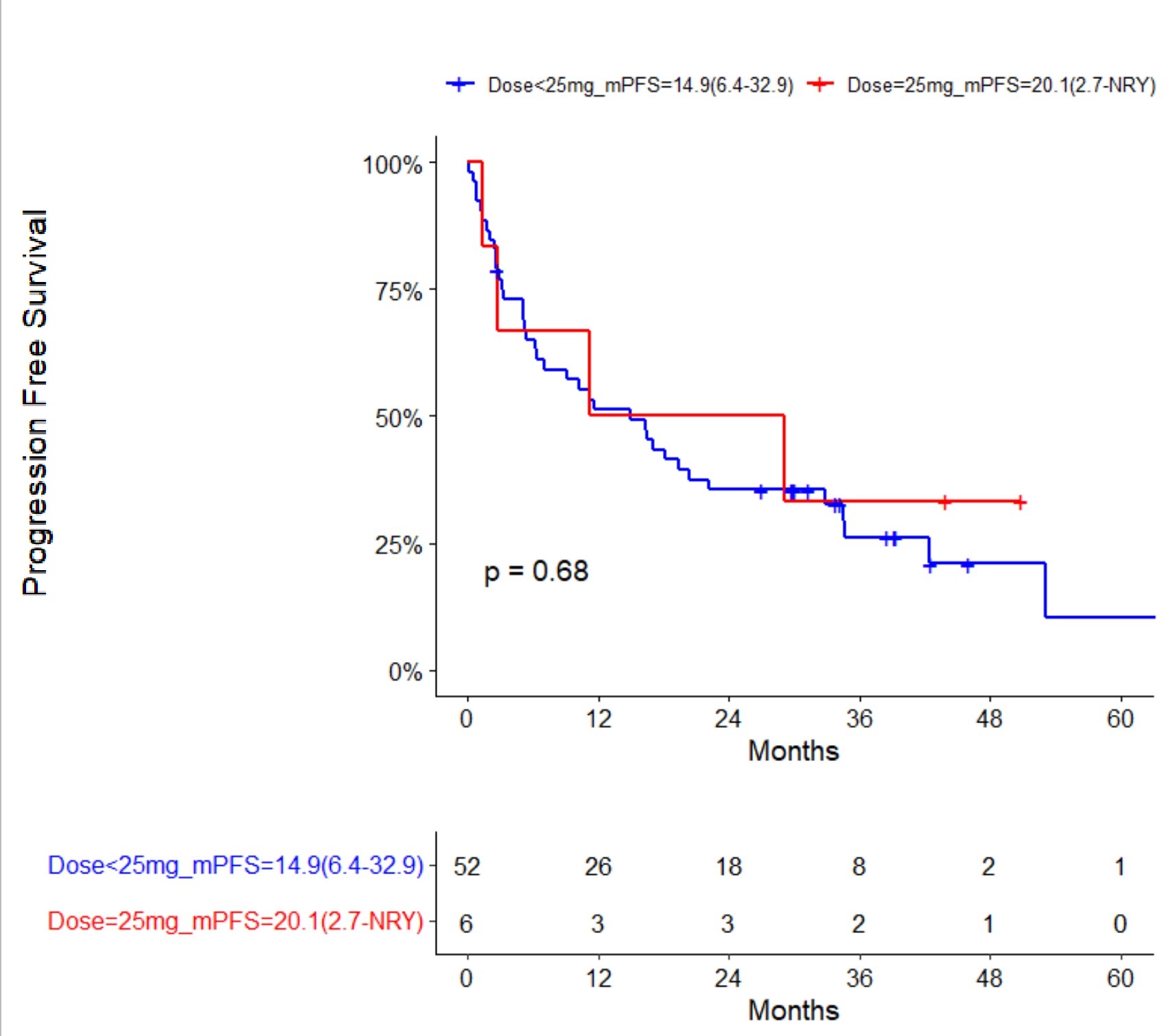

Supplement: Supplementary file 5 — Figure S3A [file CAM4-12-4357-s006.tiff]

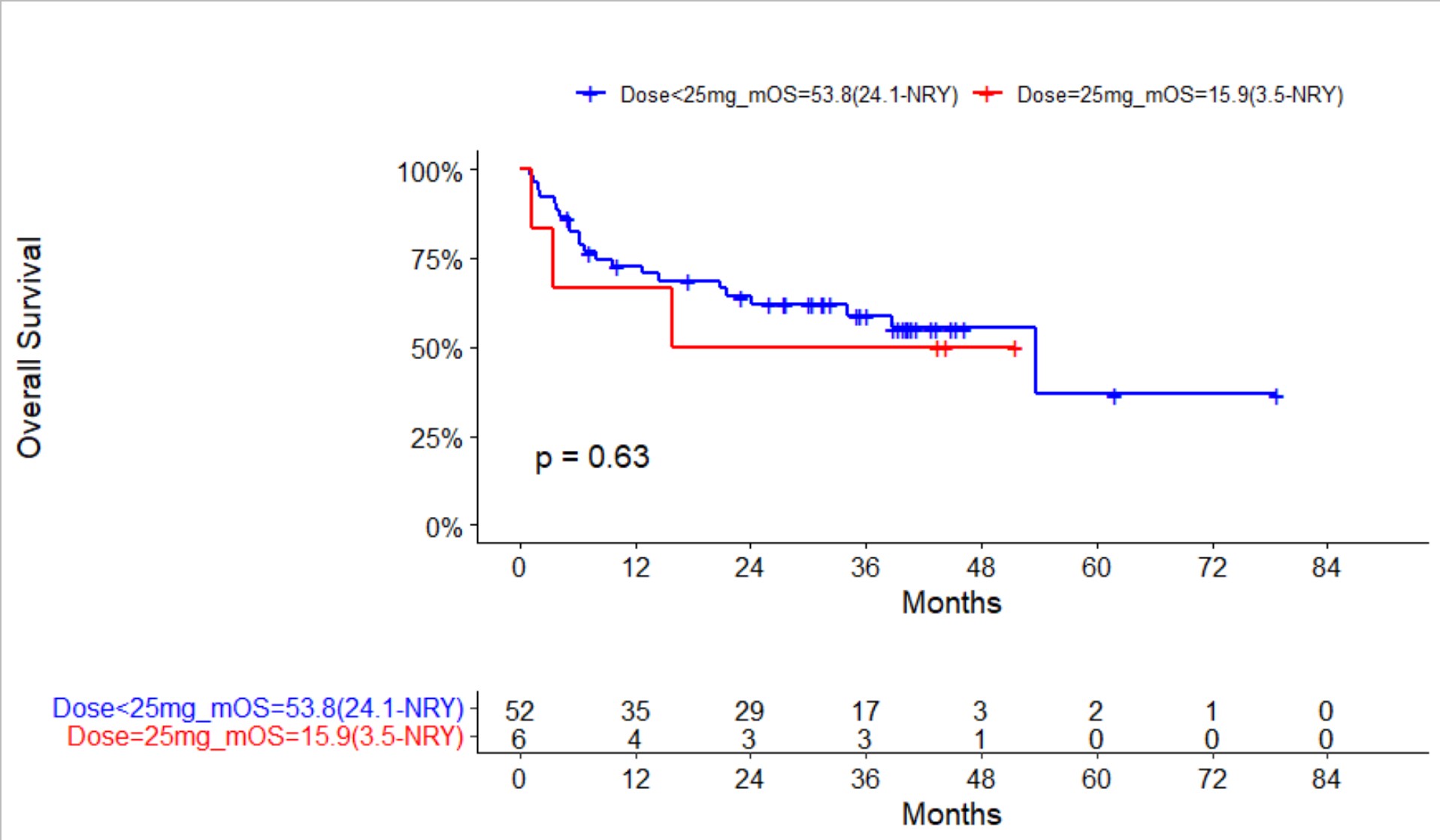

Supplement: Supplementary file 6 — Figure S3B [file CAM4-12-4357-s003.tiff]
