# Supplementary material for: Real‐world data on lenalidomide dosing and outcomes in patients newly diagnosed with multiple myeloma: Results from the Canadian Myeloma Research Group Database
Source: Cancer Med. 2022 Sep 26;12(4):4357–62. doi: 10.1002/cam4.5245 (PMC9972020; doi:10.1002/cam4.5245)
Supplement: Supplementary file 7 — Table S1 [file CAM4-12-4357-s001.docx]

**Table S1:**Lenalidomide dose modifications, duration on treatment and best responses stratified by starting lenalidomide dose

| Parameter | All patients  (N=163) | Starting dose | | | | | |
| --- | --- | --- | --- | --- | --- | --- | --- |
|  |  | **25 mg**  (n=63, 38.0%) | **20 mg**  (n=6, 3.7%) | **15 mg**  (n=42, 25.8%) | **10 mg**  (n=49, 30.1%) | **5 mg** (n=3, 1.8%) | **2.5 mg**  (n=1, 0%) |
| Age, years^#^  (Median, range) | 77  (58-94) | 74  (57-93) | 81  (75-85) | 79  (59-92) | 81  (64-94) | 77  (73-92) | 88 |
| eGFR mL/min/m/1.73 m^2#^,n (%)*  ≥ 60  <60  Unknown | 80 (58.0)  58 (42.0)  25 | 42 (87.5)  6 (12.5)  14 | 5 (100)  -  1 | 16 (42.1)  22 (57.9)  4 | 17 (38.6)  27 (61.4)  5 | -  2(100)  1 | -  1 (100)  - |
| Dose increased  (n, %) | 5 (3.0) | - | - | - | 4 (8.2) | 1 (33.3) | - |
| Dose decreased  (n, %) | 57 (35.0) | 25 (40.3) | 3 (50.0) | 17 (40.5) | 12 (24.5) | - | - |
| No change in dose  (n, %) | 101 (62.0) | 37 (59.7) | 3 (50.0) | 25 (59.5) | 33 (67.4) | 2 (66.7) | 1 (100) |
| First dose reduction timing, months  (median, range) | 7.4  (0.8-56.9) | 11.5  (0.8-56.9) | 20.6  (2.4-22.1) | 4.6  (0.9-24.0) | 4.6  (0.9-35.9) | - | - |
| Tx duration, months  (median, range) | 21.2  (0.1-113.2) | 21.8  (0.6-60.6) | 35.8  (5.6-113.2) | 18.5  (0.5-72.1) | 21.5  (0.1-82.8) | 18.5  (8.8-34.4) | 3.5 |
| ORR (>PR), n (%)* | 118 (78.1) | 47 (81.0) | 5 (83.3) | 26 (68.4) | 36 (83.7) | 1 (50.0) | 1 (100) |
| >VGPR, n (%)* | 78 (51.7) | 34 (58.6) | 4 (66.7) | 16 (42.1) | 22 (51.2) | 1 (50.0) | - |

^#^at the time of therapy initiation

*Percentage calculated for each category among patients with known information

eGFR, estimated glomerular filtration rate

Abbreviations: ORR, Overall response rate; PR, Partial response; VGPR, Very good partial response; >, equal or greater than
